# Peroxisome Maintenance Depends on De Novo Peroxisome Formation in Yeast Mutants Defective in Peroxisome Fission and Inheritance

**DOI:** 10.3390/ijms20164023

**Published:** 2019-08-17

**Authors:** Justyna P. Wróblewska, Ida J. van der Klei

**Affiliations:** Molecular Cell Biology, Groningen Biomolecular Sciences and Biotechnology Institute (BBA), University of Groningen, PO Box 11103, 9300 CC Groningen, The Netherlands

**Keywords:** peroxisome, organelle, Pex11, fission, inheritance, yeast, *Hansenula polymorpha*

## Abstract

There is an ongoing debate on how peroxisomes form: by growth and fission of pre-existing peroxisomes or de novo from another membrane. It has been proposed that, in wild type yeast cells, peroxisome fission and careful segregation of the organelles over mother cells and buds is essential for organelle maintenance. Using live cell imaging we observed that cells of the yeast *Hansenula polymorpha*, lacking the peroxisome fission protein Pex11, still show peroxisome fission and inheritance. Also, in cells of mutants without the peroxisome inheritance protein Inp2 peroxisome segregation can still occur. In contrast, peroxisome fission and inheritance were not observed in cells of a *pex11 inp2* double deletion strain. In buds of cells of this double mutant, new organelles likely appear de novo. Growth of *pex11 inp2* cells on methanol, a growth substrate that requires functional peroxisomes, is retarded relative to the wild type control. Based on these observations we conclude that in *H. polymorpha* de novo peroxisome formation is a rescue mechanism, which is less efficient than organelle fission and inheritance to maintain functional peroxisomes.

## 1. Introduction

Peroxisomes are important organelles widely distributed among eukaryotic organisms. These organelles compartmentalize a variety of specific metabolic processes [1,2,3]. Common functions include β-oxidation of fatty acids and detoxification of hydrogen peroxide. In yeast, peroxisomes are important for the metabolism of various unusual carbon and nitrogen sources, such as oleic acid, methanol, primary amines and uric acid [4]. Also, several non-metabolic functions have been identified for peroxisomes, such as antiviral innate immunity and anti-viral signaling in mammalian cells [5] and response to stress in yeast [6].

In yeast, peroxisome proliferation is repressed during growth of cells on media that do not require peroxisomal enzymes (e.g., glucose). However, their proliferation is induced upon shifting these cells to growth conditions that do require the activity of peroxisomal enzymes. The yeast *Hansenula polymorpha* can grow on methanol as a sole carbon and energy source. Upon shifting *H. polymorpha* cells from glucose- to methanol-containing medium peroxisome proliferation is strongly induced [7]. This property renders *H. polymorpha* an attractive model organism to study peroxisome formation.

There is an ongoing debate on how peroxisomes proliferate. Two different models have been proposed lately. The first one postulates that peroxisomes form de novo. This process involves targeting of peroxisomal membrane proteins (PMPs) to other organelles, such as the endoplasmic reticulum (ER) [8,9,10,11] or mitochondria [12] and their subsequent exit in vesicles, that eventually mature into normal peroxisomes, upon heterotypic fusion with other vesicles or pre-existing peroxisomes. The second model proposes that peroxisomes are semi-autonomous organelles, which multiply by growth and fission of pre-existing ones, like mitochondria [13,14,15]. In this model all cells should harbor at least one peroxisome, which is required for the formation of additional ones, when peroxisome proliferation is induced. The growth and fission model implies that during cell budding, peroxisomes should be properly partitioned over the mother cell and the newly formed daughter cell. So far, in yeast two proteins, Inp1 and Inp2, have been identified that play a role in peroxisome partitioning during budding. Inp1, a peripheral membrane protein of peroxisomes, is involved in peroxisome retention in mother cells [16,17]. Inp2 is a PMP that physically interacts with the myosin V motor protein Myo2, enabling active transport of peroxisomes via actin cables towards the developing bud [18,19,20].

Several lines of evidence support the occurrence of de novo peroxisome formation from the ER. Vesicles containing PMPs can bud from the ER as evident from in vitro budding assays [21,22]. Additionally, it was shown that reintroduction of the missing genes in yeast *pex3* and *pex19* mutants was followed by peroxisomes reappearance in these strains. As newly synthesized Pex3 was first spotted at the ER before localization at peroxisome membranes, the ER became a feasible candidate organelle in de novo biogenesis of peroxisomes [23,24]. Moreover, the ER-localized peroxins Pex30 and Pex29 have been proposed to regulate de novo biogenesis of peroxisomes at ER exit sites for pre-peroxisomal vesicles [25,26,27]. Similarly, the model of peroxisome fission and inheritance has been well documented. Many components of the fission machinery have been identified, such as Pex11, dynamin-like proteins (Vps1/Dnm1), Fis1 and Mdv1/Caf4 adaptor proteins. Peroxisome fission has been proposed to be the major pathway of peroxisome proliferation in wild type (WT) yeast cells [28]. If true, a complete block in peroxisome fission will result in a reduction in peroxisome number, ultimately leading to peroxisome deficiency in the progeny of the original mutant cell. To test this model, we analyzed mutants lacking genes involved in peroxisome fission and inheritance in *H. polymorpha*.

Our results indicate that blocking peroxisomal fission and inheritance simultaneously (in *H. polymorpha pex11 inp2* mutant cells) results in the formation of yeast buds devoid of any peroxisomal structure, in which new peroxisomes most likely form de novo. This process is relatively slow. Moreover, *pex11 inp2* cells show enhanced doubling times relative to the WT control or *pex11* or *inp2* single deletion strains on growth media that require functional peroxisomes (methanol). This suggests that peroxisome fission and inheritance are responsible for the maintenance of peroxisomes in WT cells, whereas de novo peroxisome biogenesis is a rescue mechanism that allows the formation of new peroxisomes in mutant cells devoid of pre-existing ones.

## 2. Results

### 2.1. Almost All H. polymorpha pex11 Cells Contain Peroxisomes

**Figure 1:**
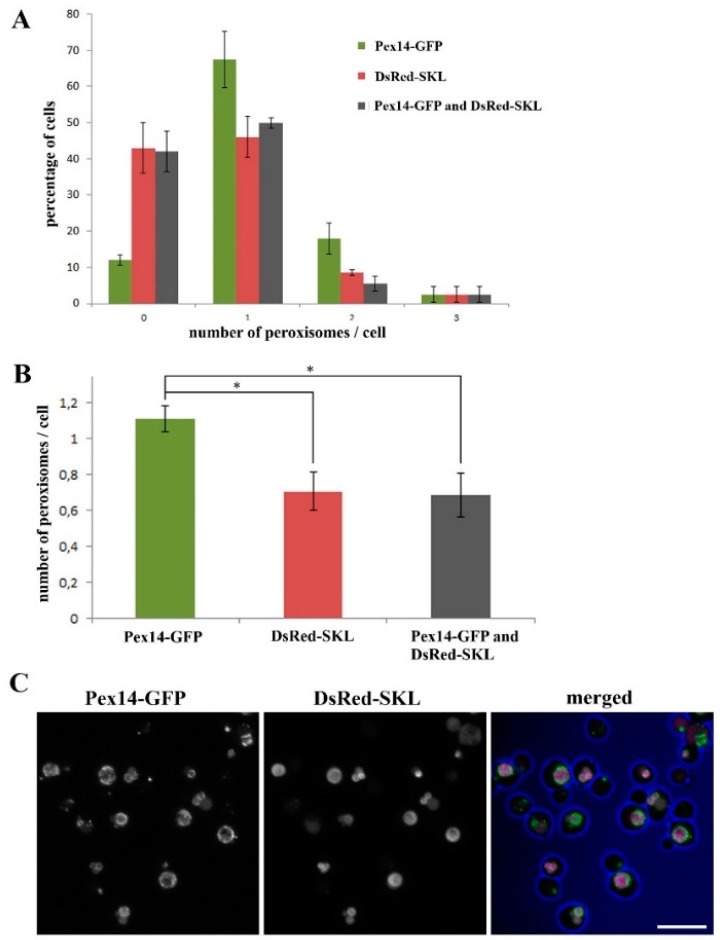
Almost all *H. polymorpha pex11* cells possess at least one peroxisome. (**A**) Frequency distributions of fluorescent spots in *H. polymorpha pex11* cells. Fluorescent structures were quantified using the indicated peroxisomal fluorescent markers. Cells were grown for 16 h on methanol containing medium. Data were obtained from two independent experiments (*n* = 2). In each experiment peroxisomes were counted in 100 cells, including buds with a diameter comprising at least 1/3 of corresponding mother cell’s diameter. The error bars represent the standard deviation. (**B**) Comparison of the average number of peroxisomes per cell detected in *pex11* cells using different fluorescent protein markers. More peroxisomes are detected when solely Pex14-GFP is used as a peroxisome marker (* *p* < 0.05). Cells were grown as indicated in Figure 1A. (**C**) Example of confocal laser scanning microscopy (CLSM) image representing *pex11* cells used for the quantification shown in (**A**) and (**B**). Blue represents cell edges. The scale bar represents 10 µm.

Previous quantitative analysis of *H. polymorpha pex11* cells, using confocal laser scanning microscopy (CLSM) and the peroxisomal membrane marker protein PMP47-GFP [29], revealed an average number of peroxisomes per cell of 0.7 and a significant fraction of cells lacking peroxisomes (56%). When using a matrix marker (DsRed-SKL) the percentage of cell lacking peroxisomes and the average number of peroxisomes per cell were similar to those obtained using PMP47-GFP as a marker (40%and 0.7 respectively; Figure 1). However, using these markers the smaller organelles may have been missed. In order to facilitate detection of all organelles, we now used Pex14-GFP as a peroxisomal marker. Pex14 has been reported to be enriched on the smaller organelles in *H. polymorpha* [30]. Indeed, using this marker the average number of organelles per cell increased to 1.1 for the *pex11* strain (Figure 1), together with a strong decrease in the percentage of cells in which no peroxisomal structure could be detected (to approximately 10%). This indicates that it is beneficial to use Pex14-GFP as a peroxisomal marker to detect peroxisomes by CLSM.

### 2.2. Peroxisomes can be Inherited to H. polymorpha pex11 Daughter Cells

**Figure 2:**
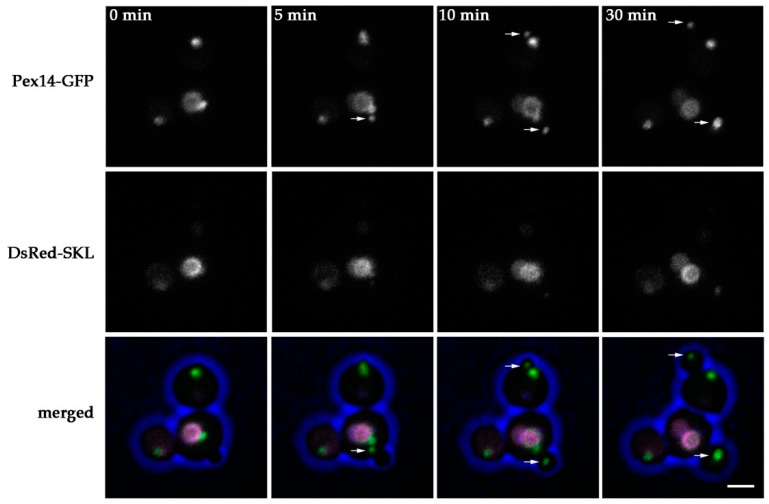
Peroxisome fission and inheritance in *H. polymorpha pex11*. Stills from a CLSM video (Supplementary Data S1) representing budding *pex11* cells producing Pex14-GFP and DsRed-SKL. Blue represents cell edges. Arrows point to Pex14-GFP containing spots that separate from a mother organelle during yeast budding and are transferred to a forming daughter cell. Images were acquired with 5 min time intervals. Scale bar represents 2 µm.

Based on the quantitative analysis, we conclude that in *pex11* cells peroxisome fission is most likely not completely blocked or compensated by de novo peroxisome formation. Moreover, newly formed buds may still inherit a peroxisome from *pex11* mother cells, explaining why almost all *pex11* cells contain peroxisomes. To test this, we performed live cell imaging of *pex11* cells producing Pex14-GFP together with the matrix marker DsRed-SKL to facilitate the detection of all peroxisomal structures. Four movies were acquired, in which in total seventeen yeast budding events were observed. In almost 50% of the budding events a Pex14-GFP spot was detected, which seems to pinch off from the mother organelle (Figure 2, Supplementary Data S1), followed by transfer to the newly forming bud. This suggests that peroxisome fission still occurs in *pex11* cells. Occasionally, a Pex14-GFP spot was transferred to the forming bud leaving the mother cell devoid of any detectable Pex14-marked peroxisomal structures. This event was followed by reappearance of a GFP fluorescent spot in the mother cell at a later stage. When no transfer to the bud was detected, a Pex14-GFP spot appeared at a later stage in the bud as well (Supplementary Data S1). Possibly, a Pex14-GFP containing membrane was still transferred to these buds, but not detected due to the low fluorescence levels, or a new Pex14-GFP containing peroxisome formed de novo. Together, our data suggest that fission and inheritance of peroxisomes still can occur in *pex11* cells, but possibly does not happen during each budding event.

### 2.3. Peroxisome Inheritance is not Detected in pex11 inp2 Cells

**Figure 3:**
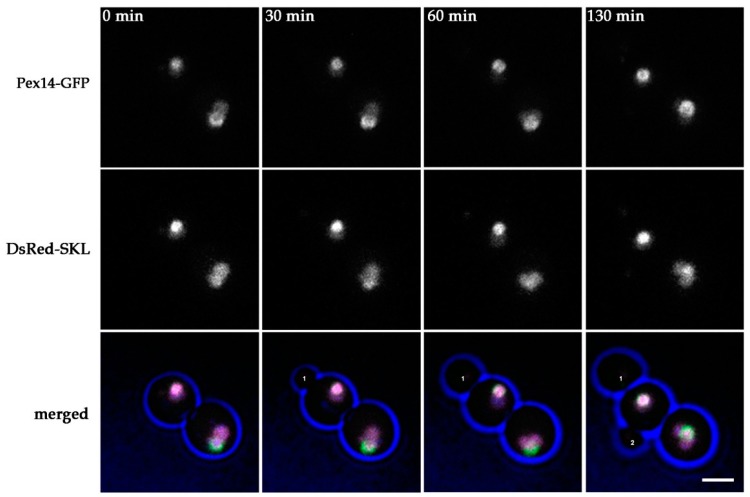
In *H. polymorpha pex11 inp2* cells peroxisome inheritance is fully blocked. Stills from a CLSM video (Supplementary Data S2) representing budding *pex11 inp2* cells producing Pex14-GFP and DsRed-SKL. Blue represents cell edges. Labels “1” and “2” mark two different daughter cells that did not inherit Pex14-GFP containing structures from the mother cell. Scale bar represents 2 µm.

Because peroxisome fission and inheritance most likely is not completely blocked in the absence of Pex11, we performed similar live cell imaging experiments using a *pex11 inp2* double mutant, in which peroxisome inheritance to the buds is expected to be blocked. In total, forty budding events were analyzed that were captured in four separate movies. In none of the forty budding events a Pex14-GFP spot was detected that moved to the bud (Figure 3, Supplementary Data S2), suggesting that peroxisome inheritance is fully blocked in this strain, resulting in bud cells devoid of peroxisomes.

### 2.4. Block of Fission and Inheritance Increases the Number of Cells Devoid of Peroxisomes

**Figure 4:**
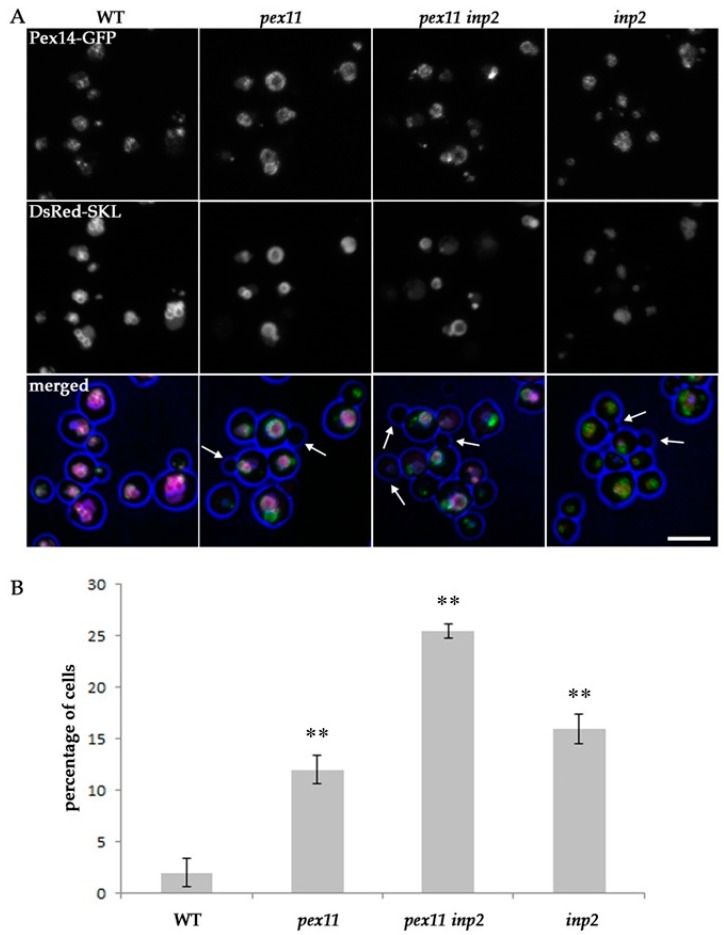
Block of fission and inheritance has an effect on the percentage of cells lacking any peroxisomal structures. (**A**) Examples of CLSM images used for the quantification shown in (**B**). Wild type (WT), *pex11*, *pex11 inp2* and *inp2* cells producing Pex14-mGFP and DsRed-SKL were grown for 16 h on methanol medium. Arrows point to the cells that lacked a Pex14-GFP spot. The scale bar represents 5 µm. (**B**) Graph representing percentage of cells lacking Pex14-GFP spots in the indicated strains. Both mother cells and buds, with a diameter comprising at least 1/3 of corresponding mother cell’s diameter, were included in the analysis. Quantitative data were obtained from two independent cultures of each strain. The percentages were calculated from 100 cells per culture. The error bars represent the standard deviation. The number of cells lacking Pex14-GFP spots was significantly higher in *pex11*, *pex11 inp2* and *inp2* strains compared to WT cells (** *p* < 0.01).

To further understand the effect of blocking peroxisome fission and/or inheritance on peroxisome abundance, we quantified peroxisome numbers in cells of WT, *pex11*, *pex11 inp2* and *inp2* strains using CLSM. All strains produced Pex14-GFP and DsRed-SKL to visualize peroxisomal structures. A cell was considered to lack any peroxisomal structure when no green fluorescent spot of Pex14-GFP could be detected. Because structures containing DsRed fluorescence, without any detectable GFP fluorescence, most likely represent vacuoles, these structures were not counted. As shown in Figure 4, the percentage of cells in which peroxisomal structures were not detected was significantly enhanced in the three mutant strains relative to the WT control. In *pex11* and *inp2* cultures, approximately one out of eight cells lacked a detectable GFP spot, whereas in the *pex11 inp2* strain one out of four cells lacked a green fluorescent spot. These results show that peroxisome fission and inheritance both contribute to peroxisome abundance in WT *H. polymorpha* cells.

### 2.5. Peroxisomes Reappear in Buds of pex11 inp2 Cells

**Figure 5:**
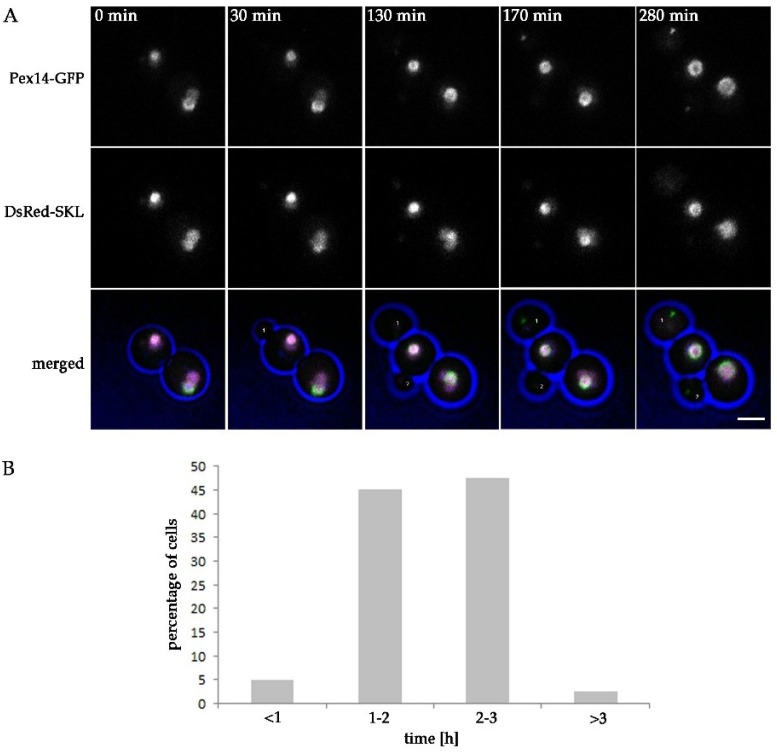
Peroxisomes re-appear in buds of the *H. polymorpha pex11 inp2* strain. (**A**) Stills from a CLSM video (Supplementary Data S2) representing budding *pex11 inp2* cells producing Pex14-GFP and DsRed-SKL. Blue represents cell edges. Numbers mark daughter cells that did not receive a Pex14-GFP spot from the mother cell and acquired it at later stages of growth. Scale bar represents 2 µm. (**B**) Time between bud formation initiation and appearance of a Pex14-GFP spot in the *pex11 inp2* buds. Initiation of bud formation was taken as a zero-time point, individually for each budding event. Forty cell budding events were included in the analysis.

Although *pex11 inp2* cells appear to be fully blocked in peroxisome inheritance, most *pex11 inp2* cells still do contain peroxisomes. This suggests that new peroxisomes form de novo in buds lacking these organelles. To check whether a Pex14-GFP spot ultimately appears in all newly formed *pex11 inp2* buds, we analyzed forty budding events by live cell imaging. The results show that in all analyzed buds a Pex14-GFP spot ultimately appeared (Figure 5, Supplementary Data S2). Quantification (Figure 5B) revealed that, in most cells, a Pex14-GFP spot appeared within 1–3 h after initiation of bud formation. However, in some cases the spot appeared within one hour or after more than three hours. These observations suggest that de novo biogenesis of peroxisomes restores the peroxisome population in buds of *pex11 inp2* cells, by a relatively slow process.

In addition, we assessed the reappearance of peroxisomes in *pex11 inp2* cells by analyzing the capacity of individual cells to grow on methanol. This experiment was based on the fact that only cells containing functional peroxisomes can grow on methanol as sole carbon source [7]. Cells from an exponential, methanol-containing batch culture were plated on a glucose-containing agar plate. After colonies appeared, the plates were replicated onto plates that contained methanol as a sole carbon source. A total of 105 colonies of the *pex11 inp2* strain were analyzed. As shown in Supplementary Data S5, all colonies grew on methanol. Hence, although in the methanol grown batch culture approximately 25% of the cells lacked a peroxisome (Figure 4), the colonies that were formed from these cells all were capable to grow on methanol. This suggests that ultimately in all newly formed buds peroxisomes are formed, possibly by de novo biogenesis.

### 2.6. The Combined Fission and Inheritance Defects of H. polymorpha pex11 inp2 Cells Increase the Doubling Time on Methanol Medium

**Figure 6:**
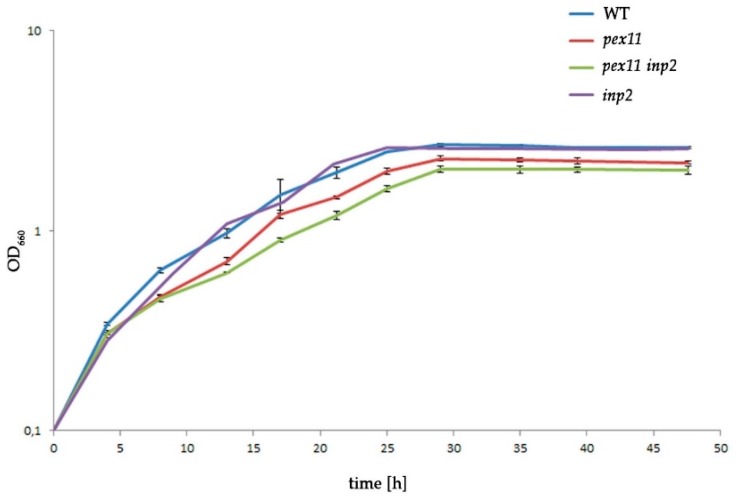
Impaired fission and inheritance affect growth of *H. polymorpha* cells. Growth curves of *H. polymorpha* WT, *pex11*, *pex11 inp2* and *inp2*. Cells were pre-cultivated on glucose medium and shifted to methanol-containing medium (T = 0 h). The densities of the cultures are expressed as optical density at 660 nm (OD_660_). The results represent mean values of three biological replicates (*n* = 3). The error bars stand for standard deviation. The OD_660_ values at 25h and 48h differed significantly between WT, *pex11* and *pex11 inp2* (*p* < 0.05) but not between WT and *inp2* cultures.

Because peroxisome-deficient *H. polymorpha* cells are unable to grow on methanol, we expected that the presence of a significant portion of cells (temporarily) lacking peroxisomes will affect growth of the cells in batch cultures containing methanol. As shown in Figure 6, *pex11* cultures showed enhanced doubling times and the final density of the culture was lower in comparison to the WT, in accordance to earlier data [17]. These effects were slightly stronger in the *pex11 inp2* double mutant. However, no differences in growth characteristics were observed between WT and the *inp2* single mutant. This result was surprising as we expected the *inp2* mutant to grow with rates comparable to *pex11*, given the similar percentages of cells without peroxisomes (see Figure 4).

### 2.7. inp2 Cells are Not Fully Defective in Peroxisome Inheritance

**Figure 7:**
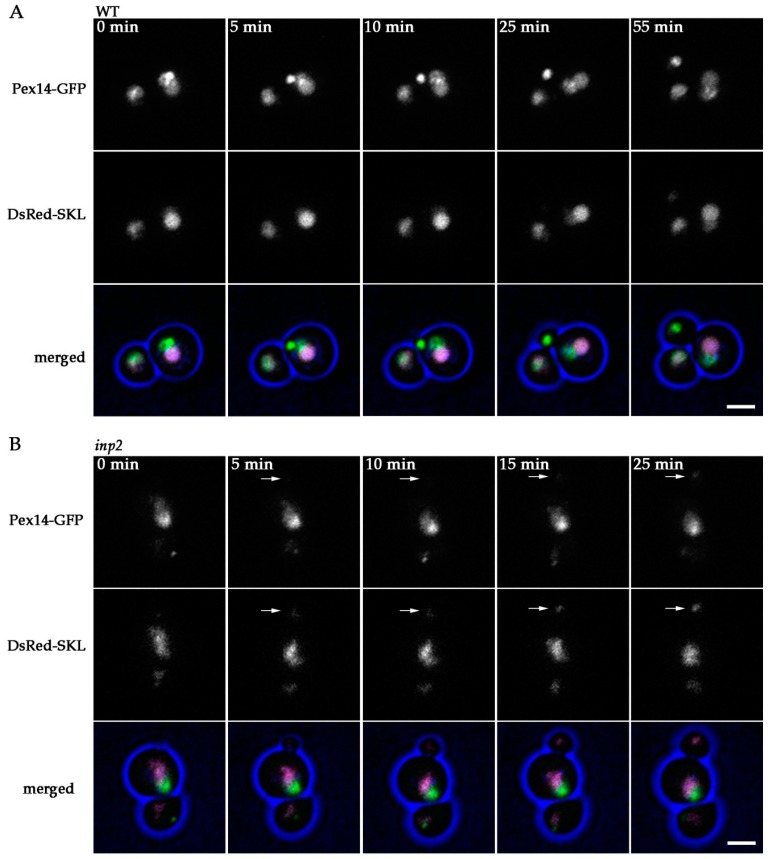
Peroxisome inheritance in *H. polymorpha* WT and *inp2* cells. Stills from a CLSM video representing budding (**A**) WT (Supplementary Data S4) and (**B**) *inp2* cells (Supplementary Data S3), producing Pex14-GFP and DsRed-SKL. Blue represents cell edges. Images were acquired with 5 min time intervals. Arrows in (**B**) point to fluorescent spots that were transferred from a mother organelle to a forming daughter cell during yeast budding. Scale bar represents 2 µm.

Even though Inp2 is a protein responsible for inheritance of peroxisomes, no growth defect was observed for *inp2* cells grown on media containing methanol (Figure 6). This suggests that in *inp2* cells still some residual peroxisome inheritance may occur. To test this, we performed live cell imaging of cells of the *inp2* single deletion strain expressing Pex14-GFP and DsRed-SKL, grown at peroxisome inducing conditions. In the WT control cells (Figure 7A, Supplementary Data S4) almost all buds received a Pex14-GFP containing structure of high fluorescence intensity from the mother cell in less than one hour after the start of bud formation. One cell budding out of nine was accompanied with transfer of a fluorescent spot of low intensity. Similar to that, during all eight cell buddings of *inp2* cells that were captured, a low intensity spot containing Pex14-GFP and DsRed-SKL was transferred from the mother cell to the bud (Figure 7B, Supplementary Data S3). Both in WT and *inp2* cells these faint spots matured into bigger ones, of higher fluorescence intensity, within 80–210 min. This indicates that inheritance of a peroxisome or a peroxisomal membrane fragment can occur in *H. polymorpha* cells, independently of Inp2.

## 3. Discussion

Here, we show that in *H. polymorpha* cells peroxisomes can multiply and inherit to daughter cells in the absence of Pex11. Also, in cells lacking the peroxisome inheritance factor Inp2, peroxisomal structures can still move to the nascent buds. However, when both Pex11 and Inp2 are absent, a substantial percentage of cells lacking peroxisomes are formed, suggesting that both fission and inheritance are blocked. These peroxisome-deficient cells ultimately become capable of growing on methanol medium, indicating that new peroxisomes form in these cells.

Our data show that peroxisomes still can divide in the absence of the key component of the peroxisome fission machinery, Pex11, which suggests that other factors, in addition to Pex11, may facilitate this process. In the *pex11* deletion mutant peroxisome fission took place only just before cell budding. It is tempting to speculate that peroxisome fission in budding and non-budding cells represent two separate processes, involving different factors and regulation mechanisms. Indeed, different peroxisome fission machineries exist. For instance, in *Saccharomyces cerevisiae* the dynamin related protein Dnm1 is responsible for fission at peroxisome inducing conditions, while the homologous protein Vps1 plays a key role in glucose-grown cells [31]. Multiplication of peroxisomes in non-budding yeast cells may serve to meet the cell’s metabolic needs. On the other hand, amplification of peroxisomes prior to yeast budding is necessary to provide nascent cells with a peroxisome. This may engage additional mechanisms to ensure undisturbed progress of the fission process.

Previously, it has been suggested that peroxisomes in *H. polymorpha* mutants lacking Pex11 are unable to undergo fission at peroxisome inducing growth conditions, however this process was studied in *pex11* cells harboring the peroxisomal membrane marker Pmp47-GFP [29]. We now analyzed peroxisome fission using DsRed-SKL as a peroxisome matrix marker, together with Pex14-GFP as membrane marker. Pex14 is more suitable as a marker for the quantification of peroxisomes in *H. polymorpha* as it also localizes to small organelles [30]. Indeed, using Pex14-GFP as a membrane marker, we detected a higher number of peroxisomal membrane structures in *pex11* cells, indicating that these were indeed most probably overlooked in previous studies.

In the *pex11* mutant peroxisome fission is not fully blocked, but most likely does not occur in all cells. Hence, upon cell budding, a subpopulation of new cells will be obtained that are devoid of peroxisomes and require de novo synthesis of this organelle.

**Figure 8:**
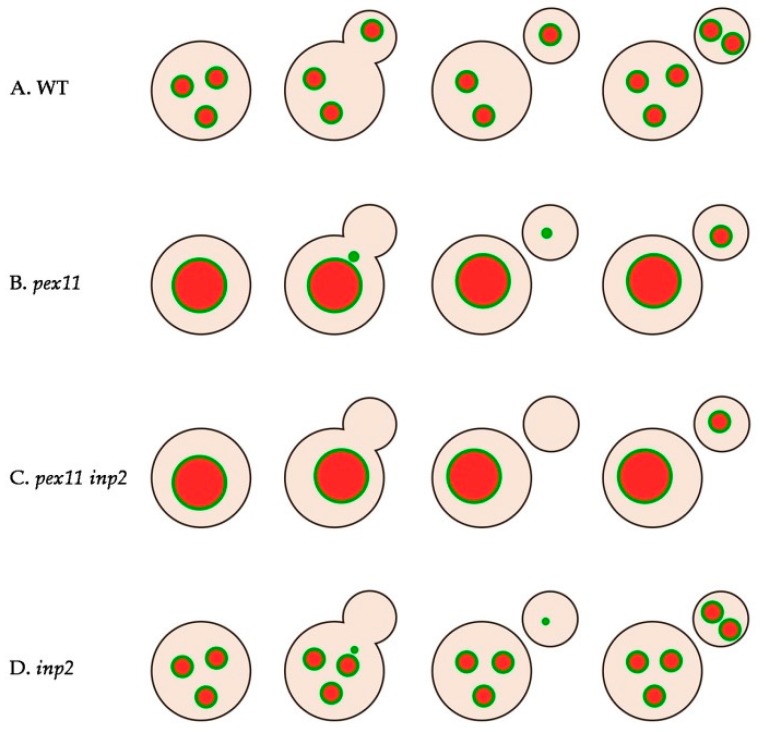
Model representing peroxisome fission and inheritance during cell budding in different strains of *H. polymorpha* grown on methanol. (**A**) In WT cells fission and inheritance are fully functional resulting in at least one of the multiple peroxisomes being transferred from the mother cell to the forming bud. (**B**) The enlarged single peroxisome in the *pex11* mutant is retained in the mother cell while a fragment of its membrane pinches off, followed by transfer to the daughter cell. This event is observed in ~50% of budding cells. The remaining ones display a situation typical for the *pex11 inp2* double mutant population (**C**), where nascent cells do not inherit any peroxisomal structure and have to form peroxisomes from a scratch. (**D**) *inp2* mutant contains multiple peroxisomes since fission is not defective in this strain. During cell budding a small organelle is transferred from the mother cell to the nascent bud, where it grows and can undergo fission.

It has been shown that at peroxisome repressing growth conditions (glucose), *H. polymorpha pex11* cells behave as *inp1* cells: peroxisomes are not retained in mother cells but instead are all transferred to the newly formed buds [17]. We here show that this peroxisome retention defect does not occur during growth of *pex11* cells on methanol. This can be due to the large size of the peroxisome during growth of *pex11* mutant cells on methanol. Our data suggest that this enlarged peroxisome stays in the mother cell, while a small portion of its membrane pinches off and then is inherited by the forming buds (Figure 2 and Figure 8B). Most likely, Inp2, which is responsible for associating peroxisomes to Myo2, is required for this process. The pulling force of Myo2 may be not sufficient to exert transfer of the entire peroxisome in *pex11* cells. Thanks to the residual fission and inheritance of small peroxisomal membrane structures, *pex11* daughter cells are provided with a membrane template that serves for the formation of a mature organelle. This is not observed anymore in the *pex11 inp2* double deletion strain, in which fission and peroxisome inheritance appears to be completely blocked (Figure 5A and Figure 8C). This points to Inp2 as a potential candidate contributing to Pex11-independent fission, at conditions that require peroxisome function.

However, it is crucial to consider the limit of detection imposed by fluorescence microscopy. We can never completely exclude that some transfer of a Pex14-GFP-containing structure occurs that is not detected because of limitations of the microscopy systems used. However, considering the large number of cells that were analyzed, it is still tempting to speculate that buds of *pex11 inp2* mutant cells initially lack any peroxisomal membrane structure and therefore are compelled to form peroxisomes from another membrane template, for instance the ER.

The ability of *pex11 inp2* cells to acquire peroxisomes was demonstrated by live cell imaging, which showed that all newly formed buds ultimately contain peroxisomes (Figure 5A). This was confirmed by the observation that all cells of a *pex11 inp2* culture are capable of forming colonies on methanol containing agar plates (Supplementary Data S5). In *pex11 inp2* batch cultures approximately every fourth cell lacks a peroxisome, but has the ability to eventually form it, and thus, metabolize methanol. This means that de novo synthesis is a feasible mode of peroxisome acquisition. This observation is in line with previous studies of peroxisome formation in *S. cerevisiae* [32] that showed that Pex11 does not play a role in de novo biogenesis. De novo formation of peroxisomes is however dependent on the other Pex11 family members, namely Pex25 and (partially) Pex27.

Why then do peroxisomes proliferate by growth and fission in WT cells in the first place? In animal cells, peroxisomes form de novo even in the presence of existing peroxisomes [33]. Some researchers pointed out the possibility that de novo peroxisome formation from the ER occurs continuously also in WT yeast cells [10,11,23,24]. Our results are not in line with this theory as we observe severe consequences on peroxisome abundance and distribution upon deletion of genes involved in peroxisome fission and inheritance. The effect of defective fission and inheritance should be not so prominent if de novo was the main mode of peroxisome proliferation.

Our results are consistent with studies by Motley and Hettema [28] using *S. cerevisiae*. These authors conducted pulse-chase experiments using GFP-PTS1 under control of the inducible *GAL1* promoter in cells that constitutively expressed HcRed-PTS1. Upon galactose induction followed by repression of the *GAL1* promotor, peroxisomes invariably showed both green and red fluorescence, indicating that peroxisomes in WT *S. cerevisiae* form exclusively by fission of pre-existing ones [28]. Additionally, these authors demonstrated that *S. cerevisiae inp2* cells lack peroxisomes as a result of a segregation defect and acquire new organelles in a slow de novo formation process. Our data indicate that in *H. polymorpha inp2* mutant de novo synthesis most likely does not take place, as peroxisome inheritance is maintained in cells devoid of Inp2 (Supplementary Data S3). This explains the unexpected observation that *H. polymorpha inp2* single mutant grew with rates similar to WT control cells, when cultivated on methanol-containing medium even though peroxisomes were not detected in a substantial percentage of the *inp2* cells. During cell budding of *inp2* mutant cells, we observed faint fluorescent structures, containing Pex14-GFP and DsRed-SKL, which were transferred from mother cells to buds (Figure 8D). Those structures probably were missed in the peroxisome quantification experiments (Figure 4) as well as in previous studies, where solely matrix protein markers were used to visualize peroxisomes and glucose was used as carbon source [28,32]. This may have led to the wrong assumption that *inp2* buds need to acquire peroxisomes de novo. However, it is also possible that Inp2-independent inheritance is a process occurring exclusively in *H. polymorpha.*

Our results indicate that, both in *H. polymorpha inp2* and in *pex11* cells, small peroxisomal fragments may be transferred to the bud, which grow into new peroxisomes. The growth difference between these two mutants can be explained by the percentage of cells that inherit peroxisomal structure. As opposed to all *inp2* cells (Supplementary Data S3), only about half of the *pex11* cells (Supplementary Data S1) acquire peroxisomal membrane fragment, imposing the need for de novo formation in the remaining *pex11* buds.

It is tempting to speculate that de novo formation of peroxisomes in cells defective in fission and/or segregation is conserved among yeast species and occurs both in cells grown on glucose [28] and at peroxisome inducing conditions. It is evident that formation of peroxisomes de novo is a time-consuming process that renders nascent buds temporarily devoid of peroxisomes (Figure 5). This has effect on the growth rate and final yields at conditions that require functional peroxisomes (Figure 6). Based on these observations, we propose that yeast cells use de novo biogenesis only as a rescue mechanism to restore the peroxisome population in cells fully devoid of peroxisomes. As evident from the analysis of the time needed for a Pex14-GFP spot to re-appear in a newly formed bud (Figure 5B), it takes 1–3 h for most of the *pex11 inp2* buds, counted from the initiation of bud formation. This may explain the slightly reduced growth rate of the double mutant strain on methanol compared to the *pex11* single deletion strain. As a result of the temporary absence of peroxisomes, peroxisomal matrix enzymes mislocalize to the cytosol, explaining the growth defect in methanol-containing medium [34].

We have to take into account that our experimental setup differed from the before-mentioned ones, therefore another plausible option is that cells can inherit a peroxisome membrane structure that mature into a normal sized peroxisome only at growth conditions where peroxisomal function is essential for growth (oleic acid/methanol). The inheritance was invariably observed in all methanol-grown *H. polymorpha inp2* cells that were analyzed, an event that has not been reported before for yeast *inp2* cells. It is possible that Inp2 is in charge of transfer of exclusively large organelles while smaller ones are inherited by another process. Examples of proteins, other than Inp2, that play a role in peroxisome inheritance are known. In *Yarrowia lipolytica*, Pex3 acts as a myosin V receptor, apart from its function in peroxisome biogenesis [35]. In *H. polymorpha*, Pex19 was implicated in peroxisome inheritance, together with Inp2 [36]. Hence, it cannot be excluded that different myosin V receptors exist to enable transport of morphologically/functionally distinct organelles at different stages of development. The inheritance event in the absence of Inp2 may also be a result of cytoplasmic streaming and occur randomly. In WT cells all the buds receive a peroxisome from the mother cell (Figure 7A and Figure 8A), however, the process it tightly controlled [18]. It cannot be excluded that WT cells can acquire a peroxisome via de novo biogenesis in case a bud accidentally does not inherit the organelle from the mother cell.

Formation of peroxisomes involving the ER membrane has been repeatedly suggested [10,11,23,24]. It may occur via formation of pre-peroxisomal vesicles containing a subset of peroxisomal membrane proteins [37]. These structures could subsequently mature into functional organelles through assembly of the importomer complex, followed by import of matrix proteins. Recent studies have shed more light on the mechanisms of de novo peroxisome formation by proposing that pre-peroxisomal vesicles (PPVs) originate from specialized regions of the ER, which are enriched in Pex30 [27]. The (ESCRT)-III (endosomal sorting complexes required for transport) complex was recently proposed to act as a scission machinery releasing peroxisomal membrane vesicles from the ER [38]. The possible engagement of mitochondria should not be excluded either [12].

## 4. Materials and Methods

### 4.1. Strains and Growth Conditions

The *H. polymorpha* strains used in this study are listed in Table 1.

*H. polymorpha* cultures were grown at 37 °C on (1) YPD media containing 1% yeast extract, 1% peptone and 1% glucose; (2) selective media containing 0.67% yeast nitrogen base without amino acids (YNB; BD Difco^TM^; Franklin Lakes, NJ, USA), supplemented with 0.5% glucose or 0.4% methanol; or (3) mineral media supplemented with 0.5% glucose or 0.4% methanol and 0.25% ammonium sulfate. When required, amino acids or uracil were added to a final concentration of 30 μg/mL. For growth on agar plates, the medium was supplemented with 2% agar. For the selection of resistant transformants YPD plates containing 100 μg/mL zeocin (InvivoGen; San Diego, CA, USA) or 300 μg/mL hygromycin B (InvivoGen) were used. For cloning purposes *Escherichia coli DH5α* was used. *E. coli* cells were grown at 37 °C in LB (1% trypton, 0.5% yeast extract, 0.5% NaCl), supplemented with 100 μg/mL ampicillin, when required.

In order to test the ability of the *pex11 inp2* cells to grow on medium containing methanol as sole carbon source, cells were pre-cultured in liquid media containing glucose and then grown in methanol-containing media. Next, cells were transferred on YND plates and then re-plated on YNM plates to check the percentage of cells that were able to grow at conditions that require peroxisome function.

### 4.2. Molecular Techniques

Plasmids and oligonucleotides used in this study are listed in Table 2 and Table 3 respectively. Polymerase and restriction enzymes were acquired from Thermo Fisher Scientific, Waltham, MA, USA). Recombinant DNA manipulations and transformation of *H. polymorpha* were performed as described before [42,43]. Preparative polymerase chain reactions (PCR) were carried out with Phusion polymerase. Initial selection of positive transformants by colony PCR was carried out using Phire polymerase. All deletions were confirmed by PCR and Southern blotting. In silico analysis of DNA sequences and construction of vector maps was carried out using Clone Manager 5 software (Scientific and Educational Software; Durham, UK).

### 4.3. Construction of Yeast Strains

The WT strain harboring Pex14-GFP and DsRed-SKL was created by introduction of the pHIPZ-Pex14-GFP plasmid linearized by the NotI restriction enzyme and the pHIPN4-DsRed-SKL linearized by SphI into the WT strain.

The *pex11* strain harboring Pex14-GFP and DsRed-SKL was created by introduction of the pHIPZ-Pex14-GFP plasmid linearized by the NotI restriction enzyme into the *pex11* (*PEX11*::*HPH*) strain expressing DsRed-SKL.

The *pex11 inp2* strain harboring Pex14-GFP and DsRed-SKL was created by deletion of *INP2* in the *pex11* (*PEX11*::*URA3*) strain expressing DsRed-SKL, using a deletion cassette containing a hygromycin resistance gene. The deletion cassette was constructed by amplification of the HPH^R^ fragment, using JWR156 and JWR157 primers and plasmid pARM001 as a template. This was followed by introduction of the pHIPZ-Pex14-GFP plasmid linearized by the NotI restriction enzyme.

The *inp2* deletion strain harboring Pex14-GFP and DsRed-SKL was created by introduction of the pHIPZ-Pex14-GFP and pHIPN4-DsRed-SKL plasmids linearized by the PstI and SphI restriction enzymes, respectively, into the *inp2* strain.

### 4.4. Fluorescence Microscopy

All images were acquired by a 100 × 1.40 NA objective using a confocal microscope - LSM800 and Zen software (Carl Zeiss AG; Oberkochen, Germany). The green fluorescence protein (GFP) signal was visualized by excitation with a 488 nm laser and the emission was detected from 490–650 nm. The DsRed signal was visualized by excitation with a 561 nm laser and the emission was detected from 535–700 nm.

For live-cell imaging, the temperature of the objective and slide was kept at 37 °C and the cells were grown on 1% agar medium. Six z-stacks were acquired for each time interval. Image analysis was carried out using ImageJ and figures were prepared using Photoshop software (Adobe; San Jose CA, USA).

### 4.5. Statistical Analysis

Significant differences between experimental groups were analyzed using GraphPad Prism software (San Diego, CA, USA). For quantitative evaluation of peroxisome numbers images of randomly chosen fields were taken as stacks of z-axis planes. The z-stacks contained twelve optical slices. The quantification was performed manually based on 100 randomly selected cells in two independent experiments. Numbers correspond to the average number of peroxisome per cell (Section 2.1) or to the average number of cells that do not contain any peroxisomal structures (Section 2.4). Significant differences between WT and different mutant strains were assessed by means of the unpaired Student’s t-test. p values <0.05 are considered as significant, and p values <0.01 are considered as highly significant.

In the analysis of the optical density at 660 nm of different strains the statistical significance between groups was tested using ANOVA and a posteriori Tukey-Kramer tests.

## Figures and Tables

**Table 1:** *H. polymorpha* strains used in this study.

Strain	Description	Reference
NCYC 495, leu1.1, ura3	Wild type	[39]
*inp2*	*INP2::URA3*	[20]
WT Pex14-GFP DsRed-SKL	pHIPZ-Pex14-GFP::*ZEO*; pHIPN4-DsRed-SKL::*NAT*	This study
*pex11* DsRed-SKL	*PEX11*::*HPH*; pHIPN4-DsRed-SKL::*NAT*	[40]
*pex11* Pex14-GFP DsRed-SKL	*PEX11*::*HPH*; HIPN4-DsRed-SKL::*NAT*; pHIPZ-Pex14-GFP::*ZEO*	This study
*pex11* DsRed-SKL	*PEX11*::*URA3*; pHIPN4-DsRed-SKL::*NAT*	[41]
*pex11 inp2* Pex14-GFP DsRed-SKL	*PEX11*::*URA3*; *INP2*::*HPH*; pHIPZ-Pex14-GFP::*ZEO*; pHIPN4-DsRed-SKL::*NAT*	This study
*inp2* Pex14-GFP DsRed-SKL	*INP2*::*URA3*; pHIPZ-Pex14-GFP::*ZEO*; pHIPN4-DsRed-SKL::*NAT*	This study

**Table 2:** Plasmids used in this study.

Name	Description	Reference
pHIPZ-Pex14-GFP	pHIPZ plasmid containing the 3′-end of the PEX14 gene fused in frame to GFP; Zeo^R^; Amp^R^	[30]
pHIPN4_DsRed-SKL	pHIPN4 plasmid containing DsRed-SKL under the control of P_AOX_; Nat^R^, Amp^R^	[30]
pARM001	pHIPH plasmid containing gene encoding C-terminal part of Pex14 fused to mCherry; Hph^R^, Amp^R^	[6]

**Table 3:** Oligonucleotides used in this study.

Name	Sequence (5′-3′)
JWR156	TTTTTATTTTATCATTTTCTATCCTCACGAGATCGCATCAAGGCACCGCTTAACCCACACACCATAGCTTCAA
JWR157	TGATGTCGAGAATCAAAAACGCTGTTGCCAGCAGCGTCGCGAGCTTCAGGCGTTTTCGACACTGGATGGC
JWR158	CGAACTGGTGGTTAAGAGCG
JWR159	GCTTTTGGCTGCGGGAACGT
JWR031	TCCTGCCAGAATTGAACTAG
JWR032	GTACGGGTAATTAACGACAC
JWR160	CACAATTGGAGCAGGACAAG
JWR161	GCCGTCGTCCTTGAAGA

## Supplementary Materials

Supplementary materials can be found at https://www.mdpi.com/1422-0067/20/16/4023/s1.
