# Supplementary figures and images for: Peroxisome Maintenance Depends on De Novo Peroxisome Formation in Yeast Mutants Defective in Peroxisome Fission and Inheritance
Source: Int J Mol Sci. 2019 Aug 17;20(16):4023. doi: 10.3390/ijms20164023 (PMC6719073; doi:10.3390/ijms20164023)

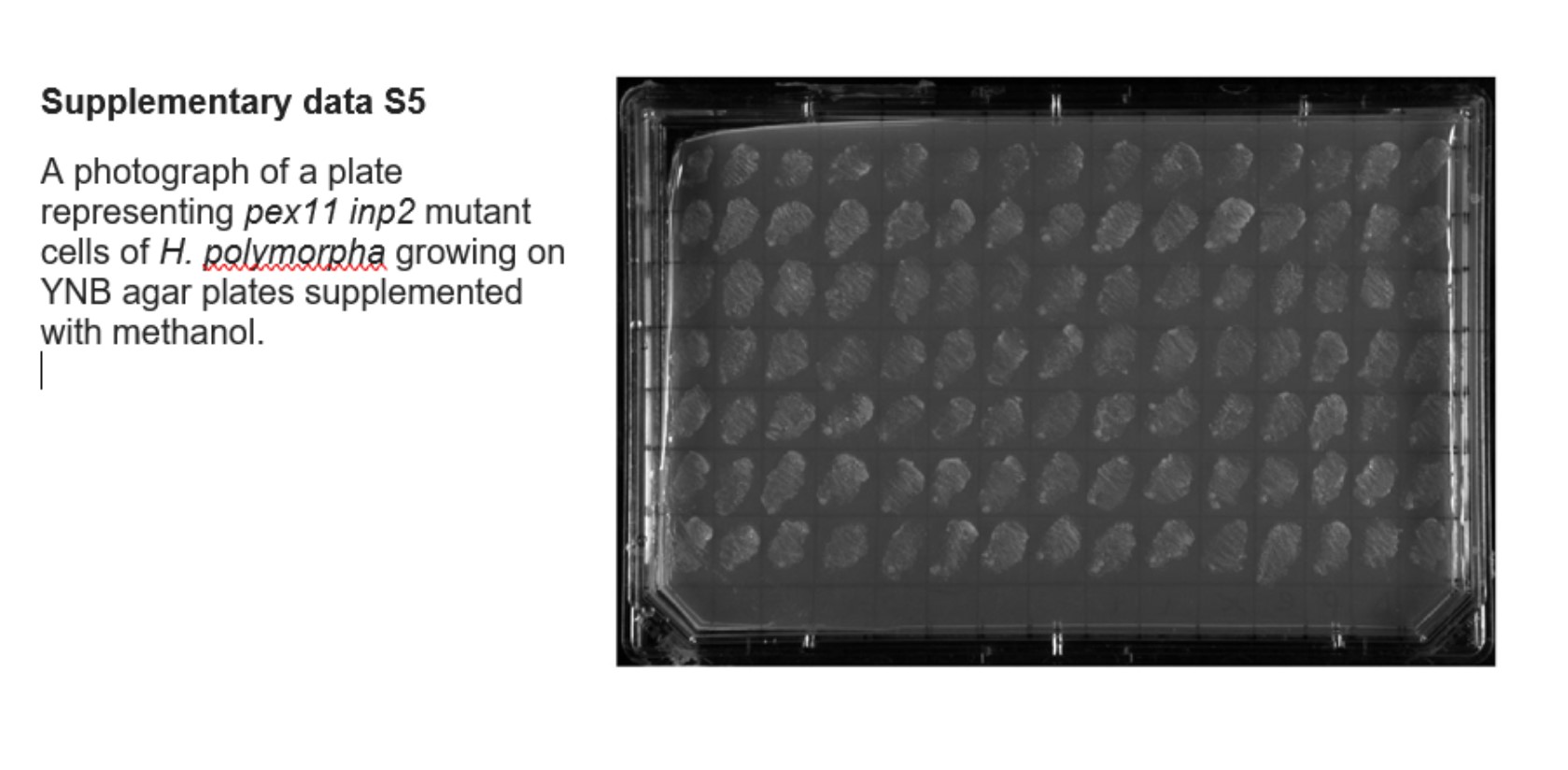

Supplement: Supplementary file 1 [file ijms-20-04023-s001.zip › Supplementary data/S5_pex11_inp2_plate.jpg]
